# Systemic Inflammation Decreases Pain Threshold in Humans In Vivo

**DOI:** 10.1371/journal.pone.0084159

**Published:** 2013-12-17

**Authors:** Moniek de Goeij, Lucas T. van Eijk, Pascal Vanelderen, Oliver H. Wilder-Smith, Kris C. Vissers, Johannes G. van der Hoeven, Matthijs Kox, Gert Jan Scheffer, Peter Pickkers

**Affiliations:** 1 Department of Anesthesiology, Pain and Palliative Medicine. RUNMC, Nijmegen, The Netherlands; 2 Department of Intensive Care Medicine, RUNMC, Nijmegen, The Netherlands; 3 The Nijmegen Institute for Infection, inflammation and Immunity. RUNMC, Nijmegen, The Netherlands; University of Arizona, United States of America

## Abstract

**Background:**

Hyperalgesia is a well recognized hallmark of disease. Pro-inflammatory cytokines have been suggested to be mainly responsible, but human data are scarce. Changes in pain threshold during systemic inflammation evoked by human endotoxemia, were evaluated with three quantitative sensory testing methods.

**Methods and Results:**

Pressure pain thresholds, electrical pain thresholds and tolerance to the cold pressor test were measured before and 2 hours after the intravenous administration of 2 ng/kg purified *E. coli* endotoxin in 27 healthy volunteers. Another 20 subjects not exposed to endotoxemia served as controls. Endotoxemia led to a rise in body temperature and inflammatory symptom scores and a rise in plasma TNF-α, IL-6, IL-10 and IL-1RA. During endotoxemia, pressure pain thresholds and electrical pain thresholds were reduced with 20±4 % and 13±3 %, respectively. In controls only a minor decrease in pressure pain thresholds (7±3 %) and no change in electrical pain thresholds occurred. Endotoxin-treated subjects experienced more pain during the cold pressor test, and fewer subjects were able to complete the cold pressor test measurement, while in controls the cold pressor test results were not altered. Peak levels and area under curves of each individual cytokine did not correlate to a change in pain threshold measured by one of the applied quantitative sensory testing techniques.

**Conclusions and Significance:**

In conclusion, this study shows that systemic inflammation elicited by the administration of endotoxin to humans, results in lowering of the pain threshold measured by 3 quantitative sensory testing techniques. The current work provides additional evidence that systemic inflammation is accompanied by changes in pain perception.

## Introduction

Pain is a major source of suffering in intensive care patients and commonly involves patients with concurrent inflammatory conditions like trauma, auto-immune diseases, or infectious diseases. Highly organized neural circuits existing in the brain and spinal cord regulate pain. These circuits in turn are influenced by a number of pathological states, including inflammation. Inflammation leads to a broad constellation of adaptive changes, called the ‘sickness response’. Features of this response include fever, increased sleep, decreased locomotion, decreased food and water intake, and hormonal changes [1]. Furthermore, the pain threshold for painful stimuli is lowered, resulting in hyperalgesia, and normally non-painful stimuli can become painful (allodynia). Hyperalgesia constitutes an underexposed clinical problem on the intensive care, which deserves specific attention, since e.g. opiates that are frequently used in everyday practice can actually worsen hyperalgesia [2]. Animal studies have shown that inflammatory cytokines contribute to the development of hyperalgesia [3-6]. However, data in humans *in vivo* are scarce.

Quantitative sensory testing (QST) provides a standardized way of pain threshold quantification, as a result of which it is increasingly used in clinical and research settings. QST comprises several techniques to measure the intensity of stimuli needed to produce specific sensory perceptions. Common stimuli employed are touch or pressure, vibration, electrical stimulation or exposure to heat or cold [7]. 

Intravenous administration of endotoxin in healthy volunteers, a well-characterized standardized model of systemic inflammation, results in an acute systemic inflammatory response characterized by high cytokine levels and flu-like symptoms [8]. In the present study, pain thresholds, measured by three separate methods of QST, were investigated before and after the administration of 2 ng/kg of *E. Coli* endotoxin in healthy volunteers to quantify the difference in pain perception caused by systemic inflammation. Additionally, we assessed the relation between circulating levels of inflammatory cytokines and pain thresholds to gain further insight in the pathophysiological mechanisms linking inflammation and pain perception. 

## Materials and Methods

### Design

The study consisted of two parts. In the first part 27 healthy young males were subjected to experimental human endotoxemia, during which quantitative sensory testing was performed before and two hours after endotoxin administration. In the second part, 20 healthy young males underwent the same QST measurement regimen without endotoxemia, to identify any possible carry-over or time-dependent effects (control experiments). The study was not designed as a randomized controlled trial, as it was considered unethical to expose subjects in the control experiments to the risks and discomfort of the placement of an arterial and venous catheter, while no fluid was administered and no blood was sampled from them. 

### Experimental human endotoxemia

The study was approved by the local ethics committee Commissie Mensgebonden Onderzoek (CMO) regio Arnhem – Nijmegen, The Netherlands and the study was conducted according to the principles expressed in the Declaration of Helsinki. After written informed consent was obtained, 27 healthy male volunteers participated in the endotoxemia treated study group, which was part of a larger endotoxemia trial (NCT 01349699). Pre-study screening revealed no abnormalities in medical history, physical examination, routine laboratory tests and ECG. At 7:30 AM subjects were admitted to the research unit of our intensive care department. A cannula was placed in an antecubital vein to permit the infusion of 1.5 litre of 2.5%glucose/0.45% saline solution in 1 hour prior to endotoxin administration, followed by 150 ml/h until 6 h after endotoxin infusion and 75 ml/h until the end of the experiment to ensure optimal hydration [9]. A 5-lead ECG and an arterial catheter in the brachial artery of preferably the non-dominant arm, enabled continuous monitoring of hemodynamics as well as the regular drawing of blood for cytokine measurements. At T=0 h, 2 ng/kg U.S. Reference *Escherichia coli* endotoxin (E. coli O:113, Clinical Center Reference Endotoxin, National Institute of Health, Bethesda, USA) was administered intravenously over 1 minute. Subjects were asked to rate each of their inflammatory symptoms (headache, backache, muscle ache, shivering and nausea) on a 0–5 Likert scale every 30 minutes following endotoxin administration. Also, body temperature was taken every 30 minutes throughout the experiment using an automatic infrared tympanic thermometer (Genius 2, Tyco healthcare group LP, Mansfield, MA, USA). EDTA anti-coagulated blood for the determination of IL-6, TNF-α, IL-10 and IL-1RA was drawn one hour before endotoxin administration, and at t=0, 0.5, 1, 1.5, 2, 3, 4, 6 and 8 hours afterwards. Blood samples were immediately centrifuged at 2.000 g for 10 minutes at 4°C and supernatants were stored at –80 °C until batch wise determination of cytokine concentrations using a simultaneous Luminex assay according to the manufacturer’s instructions (Bio-plex cytokine assay; Bio-Rad, Hercules, California, USA). 

### Quantitative sensory testing

Somatic Pressure Pain Thresholds (PPT) and Electrical Pain Thresholds (EPT) were determined in dermatome C5, T10 and L3, one hour before and two hours after LPS administration. Measurements in dermatome C5 took place at the site of the greater tubercle of the upper arm, in dermatome T10 at the location of the iliac crest, and in L3 at the vastus lateralis muscle, half way the upper leg. All measurements were carried out contra-laterally to the side where the arterial cannula was placed. The subjects were asked to indicate thresholds for pain detection (stimulus just becoming painful), after which the stimulus was interrupted immediately. Both electrical and pressure pain thresholds were determined 3 times consecutively in each dermatome. The instructions given to the subjects were repeated after every 3 stimuli. All tests were performed in a highly standardized fashion by 2 members of our group to minimize interobserver variability. PPT measurement was performed by gradually increasing pressure with a pressure algometer (Somedic, Hörby, Sweden) until the subject indicated to experience the applied pressure as pain by saying ‘stop’. 

Electrical stimuli were administered transcutaneously in a gradually increasing mode (JNI Biomedical APS, Aalborg, Denmark) via self-adhesive electrodes. The current was gradually increased from 0 to 50 milliamperes until the subject ended the measurement himself by lifting his thumb from a metal handle at the moment the feeling just started to get painful, thereby automatically stopping the electric stimulus. For both PPT and EPT the measurements in different dermatomes were analysed separately, but to give a more general estimate of the pain threshold also a mean value of the three dermatomes was calculated which will be referred to as ‘combined PPT’ and ‘combined EPT’.

The Cold Pressor Test (CPT) was carried out as previously described [10]. Briefly, one hand of the subject was submerged to the wrist in water with melting crushed ice (0 °C). From the start of ice immersion, the subject was asked to rate the pain severity on a 0-10 Numeric Rating Scale (NRS) every 10 seconds. The subject was free to remove his hand from the ice water at any time by himself if he could not tolerate the cold any longer. The measurement was stopped by the investigators after 90 seconds or if the subject rated the pain severity as 10. Both the NRS scores as well as the time to removal of the hand (latency) from the ice water were analyzed. 

### Control experiments

To correct for possible time-dependent effects or the development of tolerance to the pain stimuli, 20 healthy subjects were subjected to QST measurements without administration of endotoxin. The protocol and timing of the QST measurements were identical to the measurements in the endotoxin-treated subjects, but subjects did not fast 12 hours before the start of the experiments, did not receive a venous or arterial cannula, no hydration fluid was administered, and no blood samples were drawn. 

### Statistical analysis

For the PPT and EPT, the average of three consecutive threshold measurements was calculated at each time point. Students paired T-test was used to compare mean pain thresholds within subjects. Correlation analysis was performed using Spearman rank R correlation or Pearson’s R correlation according to the distribution of the parameters tested (Spearman rank R for change in pain threshold (i.e. ΔPT = PT _after_–PT _before_) versus cytokine induction, and Pearson’s R for Correlations between the different QST methods). Differences in tolerance to the cold pressor test were evaluated with McNemar’s test for paired comparisons for binominal data, survival analyses using the Log-rank test and repeated measures two- way ANOVA (interaction term). Differences in proportions were analyzed with Fisher’s exact test. Statistical calculations were performed using Graphpad Prism V5.0 (Graphpad software) and SPSS 18.0 (PASW Statistics). Data are represented as mean ± SEM, unless otherwise stated. Because of the anticipated large variation in baseline pain perception between subjects, data were analyzed as % change from baseline per subject. Results were considered statistically significant at P<0.05.

## Results

### Baseline characteristics

There were no significant differences in demographic characteristics between the subjects in the endotoxin treated group (n=27, age 22±0.6 years, BMI 22.8±0.4 kg/m^2^) and control subjects (n=20, age 22±0.5 years, BMI 22.2±0.7 kg/m^2^). All subjects were of European descent. There was a difference in baseline pain perception between controls and subjects; Baseline PPT was significantly higher in the endotoxin-treated group (Combined PPT 701±49 versus 491±46 N, p=0.004), while the baseline EPT was similar in both groups (Combined EPT 15.5±1.4 versus 12.1±1.4 mV, p=0.1). Also, no difference in baseline tolerance to the cold pressure test was observed, the % of subjects that could tolerate the ice immersion for the entire duration of the measurement was 63 % (LPS group) and 50 % (control group), p=0.77. 

### Experimental human endotoxemia

**Figure 1:**
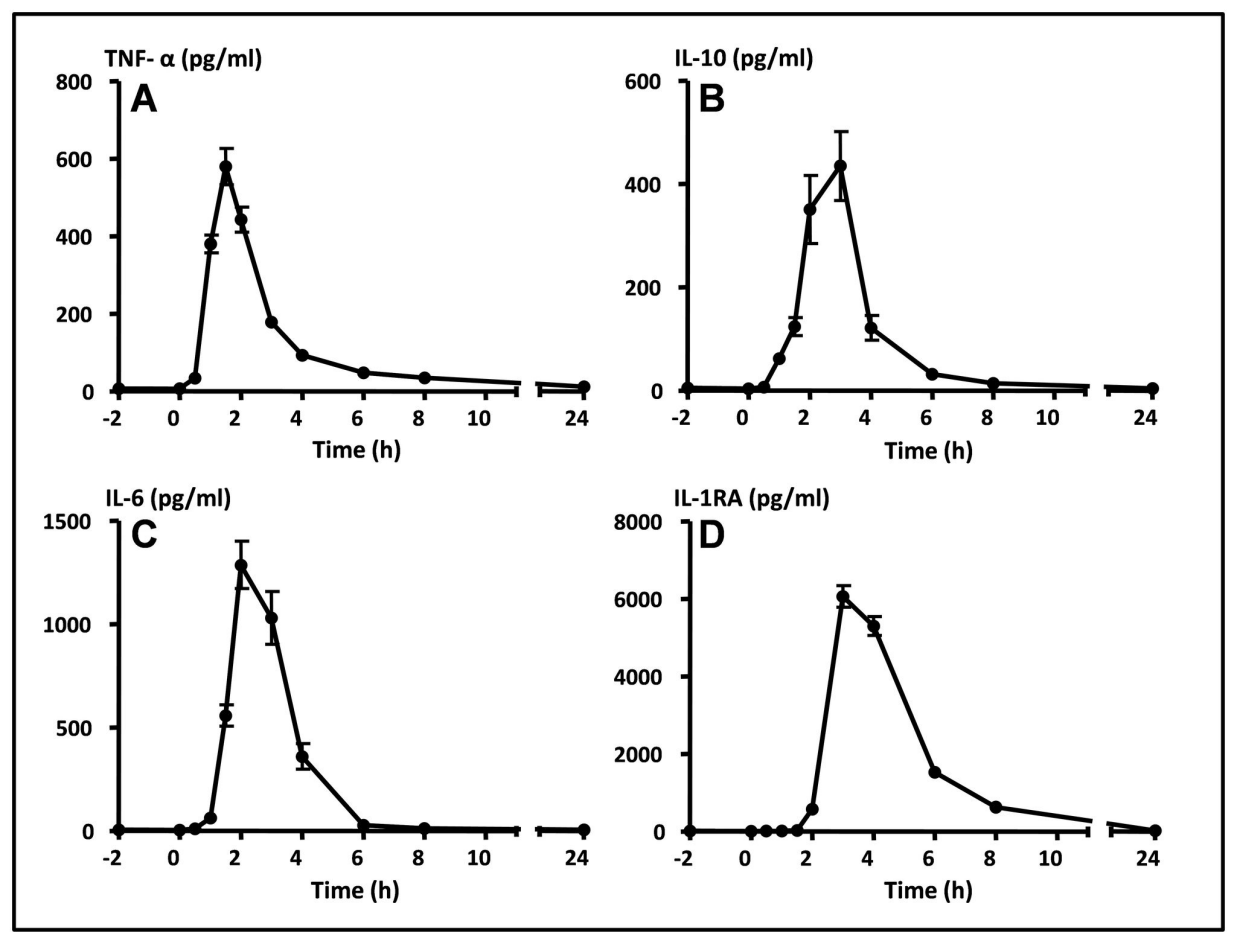
Plasma cytokine levels during human endotoxemia. Data expressed as mean±SEM. At T=0 h endotoxin was administered. A: TNF-α, B: IL-10, C: IL-6, D: IL-1RA. h: hours.

LPS administration caused a marked inflammatory response characterized by flu-like symptoms, an increase in body temperature of 2.0±0.1 °C and a marked rise in circulating cytokines in all 27 subjects. Symptoms ranging from headache, nausea, backache, muscle ache and shivering first occurred around 1 hour after endotoxin administration and peaked at T=90 minutes. Thereafter symptoms gradually declined and at discharge (T=9 h after endotoxin administration) all subjects had fully recovered. Peak levels were detected for TNF-α at 1.5 h and IL-6 at 2 h, measuring 580±47 pg/ml and 1286±114 pg/ml, respectively, while IL-10 (435±67 pg/ml) and IL-1RA (6063±282 pg/ml) peaked at 3 h (Figure 1A-D). 

### Quantitative sensory testing

**Figure 2:**
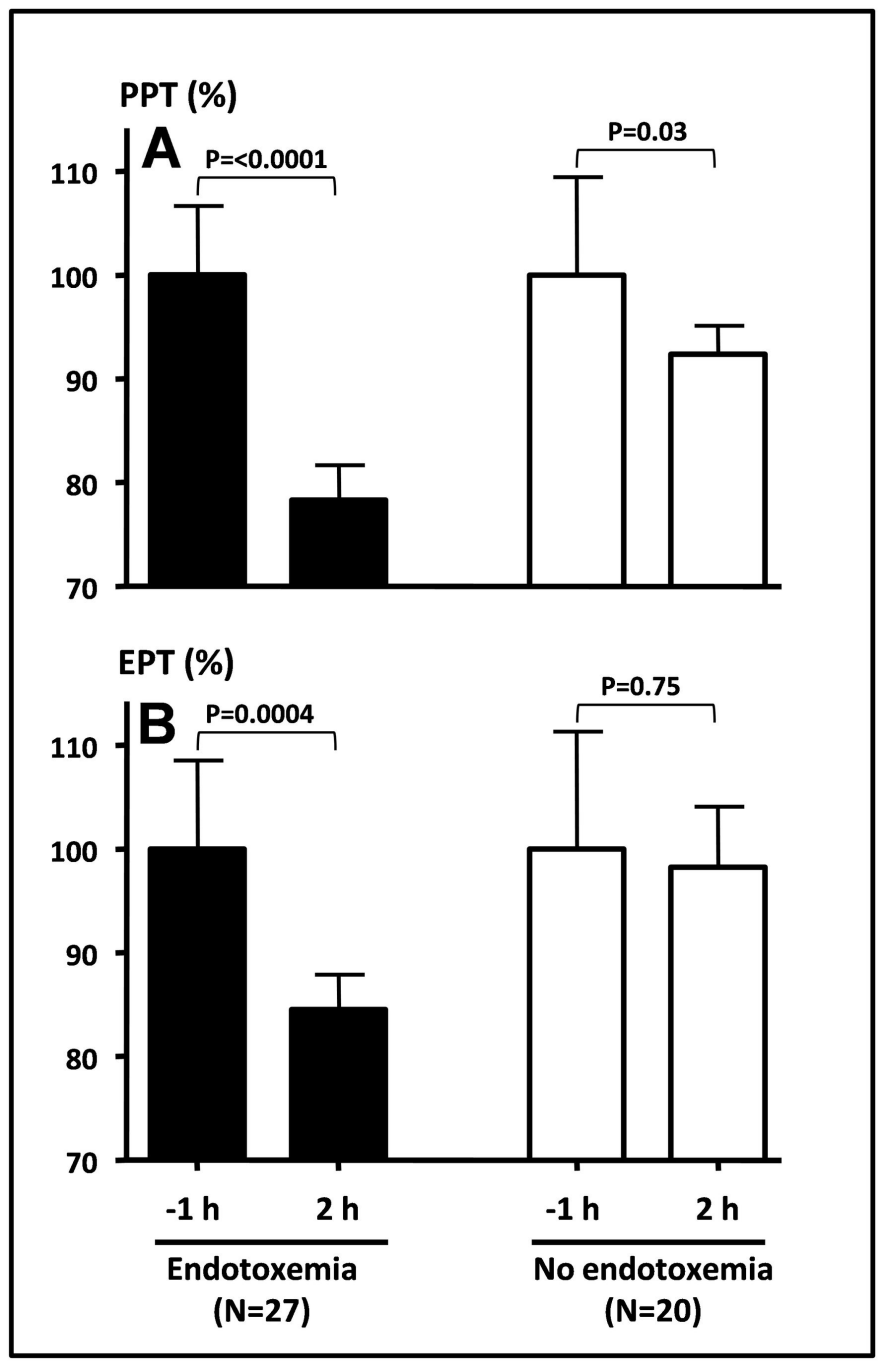
Relative change in pain thresholds during human endotoxemia. A: combined pain pressure threshold (PPT) and B: combined electrical pain threshold (EPT) measured 2 hours after endotoxin administration. Values are depicted as percentage change from baseline (-1 h), where baseline was set at 100%. White bars: control subjects, black bars: endotoxin treated subjects. Data expressed as mean±SEM. h: hours.

Pain thresholds were tested 1 hour before and 2 hours after LPS administration (Figure 2A-B, Table 1). In the endotoxin treated group a significant decrease in PPTs was noted at T=2h for each dermatome separately (C5: -20±5 %, T10: -15±5 %, L3: -23±4 %) as well as for the combined PPT (-20±4 %). In control subjects, a statistically significant, but less pronounced reduction in PPT at T=2 h was observed in dermatome C5 (-17±4 %) and combined PPT (-7±3 %). 

**Table 1:** Pain thresholds at T=-1 and T=2.

	**Endotoxemia (N=27)**					**No endotoxemia (N=20)**			
	**T=-1 hr**	**T=2 h**	**Delta**	**P-value**		**T=-1 hr**	**T=2 h**	**Delta**	**P-value**
**PPT (N)**									
C5	636±45	501±44	-136±37	0.001		483±52	402±48	-81±22	0.002
T10	639±50	534±48	-105±34	0.005		433±40	430±46	-3±14	0.85
L3	828±68	612±51	-216±43	<0.0001		556±53	545±62	-11±30	0.72
**Combined**	**701±49**	**549±45**	**-152±27**	**<0.0001**		**491±46**	**459±49**	**-31±14**	**0.03**
**EPT (mAmp)**									
C5	15.5±1.7	12.9±1.4	-2.6±1.2	0.046		12.8±1.3	12.1±1.4	-0.7±0.7	0.31
T10	17.7±1.9	14.6±1.4	-3.1±0.9	0.002		11.8±1.6	11.7±1.7	-0.1±1.4	0.94
L3	13.3±1.2	11.0±1.1	-2.3±0.6	0.0004		11.8±1.6	11.9±1.7	0.1±0.6	0.86
**Combined**	**15.5±1.4**	**12.9±1.2**	**-2.6±0.7**	**0.0004**		**12.1±1.4**	**11.9±1.5**	**-0.2±0.7**	**0.75**

PPT: Pressure Pain Threshold. EPT: Electrical Pain Threshold. N: newton. mAmp: milliamperes

Electrical pain thresholds were significantly decreased in the endotoxin treated group (C5: -8±7 %, T10: -14±4 %, L3: -16±3 %, Combined: -13±3 %), but no significant changes were observed in control subjects. 

**Figure 3:**
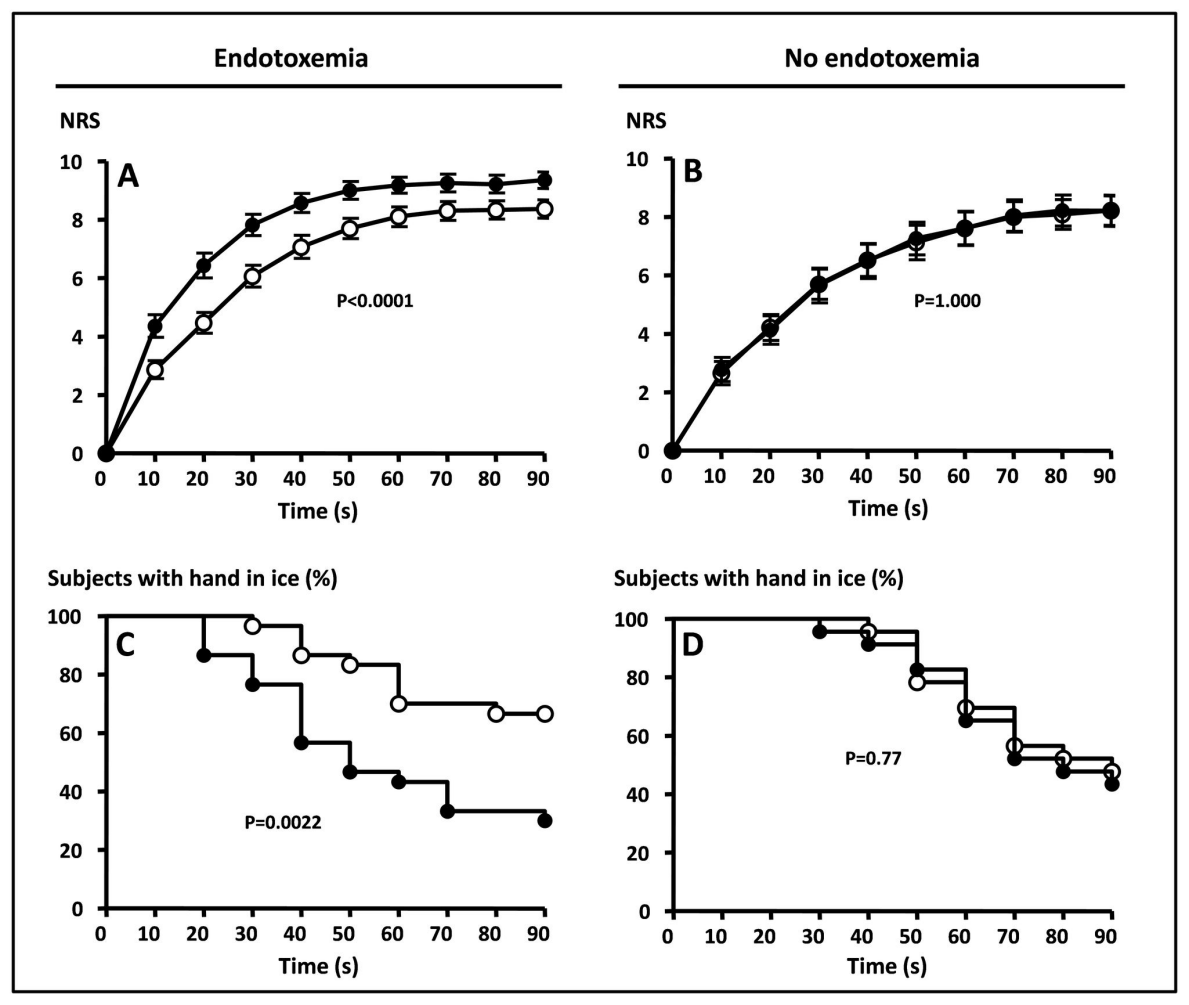
Change in tolerance to the cold pressor test during human endotoxemia. A and B: Amount of discomfort in response to immersion of one hand in ice water, rated on a 0-10 numeric rating scale (NRS). C and D: Percentage of subjects with hand in ice water. Open circles: . T=-1 hour (before endotoxin treatment), black dots: T=2 hours, (after endotoxin treatment). A and C: results in endotoxin treated group, B and D: results in control group.

Subjective pain perception measured by NRS scores in response to the cold pressor test was highly reproducible (Figure 3A-B) showing identical curves for the measurements at t=-1 and T=2 h in control subjects. Two hours after endotoxin administration, significantly more pain was reported during the cold pressor test (p<0.0001). Also, the period that subjects could withstand the exposure to cold was significantly decreased after endotoxin administration, only 26 % of the subjects completed the 90 second duration of the measurement during endotoxemia compared to 63% at T=-1 h (Figure 3C). The mean time to withdrawal from the ice was 76±4 seconds before endotoxin treatment and 54±5 seconds after (Figure 3D, p=0.0001). In control subjects there was no difference between the two measurements (55 % fulfilled the measurement at T=-1 hr versus 50 % at T=2 h, p=1.00). Mean time to withdrawal was 77±4 seconds at T=-1 and 75±4 seconds at T=2 h, p=0.51). 

### Correlation analyses

There was a good correlation between pain threshold determinations on different dermatomes and a modest, though statistically significant correlation between pain thresholds measured by PPT and EPT (Combined PPT vs Combined EPT: r=0.43, p<0.0001). Correlation analyses revealed no significant correlations between cytokine levels and changes in PPT or EPT (Table 2), except for weak correlations of peak IL-10 and delta PPT C5, IL-10 at T=2 h and delta PPT C5 and IL-6 at T=2 h and delta CPT. All these correlations lost significance following Bonferroni’s correction for multiple testing. Apart from the absence of clinically relevant correlations between cytokines and differences in pain thresholds, also no correlations between total symptom score or separate symptom scores (headache, backache, muscle ache, shivering and nausea) and changes in EPT, PPT or tolerance to CPT were found (data not shown). 

**Table 2:** Spearman correlations of changes in pain thresholds and cytokines.

		**TNF-α**						**IL-6**					**IL-10**					**IL-1RA**			
		**Peak**	**Value at t=2 h**					**Peak**	**Value at t=2 h**				**Peak**		**Value at t=2 h**			**Peak**		**Value at t=2 h**	
**ΔPPT (N)**																				
C5	r	0.187	0.013					-0.062	-0.111				0.455 *		0.412 *			0.064		0.100	
	P-value	0.342	0.949					0.755	0.575				0.015		0.029			0.746		0.614	
T10	r	0.017	-0.059					0.164	0.102				-0.034		-0.092			-0.299		0.002	
	P-value	0.932	0.767					0.404	0.606				0.864		0.641			0.122		0.993	
L3	r	-0.083	-0.167					0.121	0.144				0.030		0.067			0.050		-0.044	
	P-value	0.674	0.394					0.540	0.465				0.879		0.736			0.799		0.823	
**Combined**	r	0.155	0.056					0.200	0.173				0.223		0.164			0.026		0.093	
	P-value	0.430	0.776					0.308	0.379				0.255		0.404			0.894		0.638	
**ΔEPT (mAmp)**																					
C5	r	0.218	0.131					-0.003	-0.129				-0.045		-0.094			-0.286		-0.265	
	P-value	0.275	0.516					0.988	0.522				0.825		0.641			0.148		0.181	
T10	r	-0.128	0.052					0.053	-0.020				-0.181		-0.184			-0.009		-0.380	
	P-value	0.525	0.797					0.792	0.919				0.365		0.357			0.965		0.051	
L3	r	0.194	0.170					-0.022	-0.119				0.009		-0.089			-0.093		-0.245	
	P-value	0.333	0.398					0.914	0.554				0.963		0.660			0.646		0.219	
**Combined**	r	0.194	0.170					-0.022	-0.119				0.009		-0.089			-0.093		-0.245	
	P-value	0.333	0.398					0.914	0.554				0.963		0.660			0.646		0.219	
**ΔCPT (NRS)**																				
	r	-0.038	-0.063					0.293	0.415 *				0.102		0.166			0.249		0.248	
	P-value	0.848	0.749					0.130	0.028				0.606		0.399			0.201		0.203	

Δ: Delta. Pressure Pain Threshold. EPT: Electrical Pain Threshold. CPT: Cold Pressor Test. NRS: Numeric Rating Scale. N: Newton. mAmp: milliamperes. *:p<0.05

## Discussion

This study reports a clear change in pain perception during human experimental endotoxemia, and supports previous findings that suggest a close interrelation between the immune response and hyperalgesia. A profound difference in pain perception was found 2 hours after endotoxin administration which was not found in a group of subjects that did not receive endotoxin. Although human endotoxemia is a well–validated, standardized model of inflammation suitable to find possible correlations between cytokine release and altered pain thresholds, no such direct correlations were found. 

It has been long recognized that substances released by immune cells can modulate pain perception. The modulation of pain perception is part of the brain-mediated sickness response that enhances host survival. Pro-inflammatory cytokines are thought to initiate this response as they are the key mediators of immune to brain communication [11,12]. These cytokines can be produced locally at the site of tissue injury, leading to a local change in pain threshold. However, in the case of systemic inflammation, cytokines can induce a generalized change in pain perception. These cytokines act on signal transduction at the peripheral nerve terminals, along the nerve bundles, within the spinal cord and within the brain. In addition they can activate the vagus nerve which is also involved in pain perception and the sickness response [13]. 

IL-1 is regarded as the most important and potent cytokine in modulation of pain perception. Hyperalgesia can (at least in part) be blocked by anti-IL-1 directed therapies, while other pro-inflammatory cytokines such as TNF-alpha, and IL-6 are thought to influence pain perception mainly through IL-1 induction, illustrated by the fact that hyperalgesia caused by pro-inflammatory cytokines other than IL-1 is attenuated by blocking IL-1 signalling [14,15]. In contrast with the hyperalgesic effects of pro-inflammatory cytokines, the anti-inflammatory cytokine IL-1 receptor antagonist (IL-1RA) and IL-10 have shown to increase pain thresholds when administered intrathecally [16-19]. Not only systemic production of IL-1 is associated with hyperalgesia, also de novo production and release of IL-1 in the brain and the dorsal ganglia of the spinal cord are involved in sickness-induced hyperalgesia [20]. In addition, nerve growth factor (NGF) may represent a final common pathway, as NGF is synergistically induced by both IL-1 and TNF- alpha [21,22]. As IL-1 plays a pivotal role in changes of pain perception, it appears logical to measure IL-1 production in the current study as well. However, during human endotoxemia plasma IL-1 is not or only marginally elevated [23]. In contrast, IL-1RA, the natural inhibitor of IL-1, is markedly elevated after endotoxin administration, suggesting that there is a significant induction of IL-1 within the tissues, which is not reflected in the circulation, but which could modulate pain perception. This may be a explanation for the fact that no correlation between changes in pain thresholds and measured circulating cytokines was found. An alternative explanation is that the effects of bacterial-derived inflammatory stimuli on pain perception are not mediated by inflammatory cytokines but by these bacterial products themselves, as hyperalgesia in mice was recently shown to be dependent on bacterial load rather than immune activation [24]. In agreement, bacterial products, including LPS, can directly activate nociceptor neurons [24,25]. 

Systemic inflammation has previously been shown to alter pain perception in animals and humans. Recently, Benson and colleagues reported altered pain perception after the administration of a very low dose of endotoxin (0.4 ng/kg) to healthy volunteers, mimicking low grade systemic inflammation [26]. The current work provides additional evidence that systemic inflammation is accompanied by changes in pain perception. However, in contrast to the study of Benson et al. [26] no correlations were found between plasma levels of cytokines and changes in pain perception. A possible explanation might be that Benson et al. [26] used a 5 times lower dose of endotoxin, only inducing a mild inflammatory reaction. Furthermore, the reported correlations are difficult to interpret: similarly to the present study, no correlations between changes in pain thresholds and peak or area under curve cytokine levels were found. The only significant correlations were found at time points either before or after peak levels of the respective cytokines. This implies that a type-1 error may have occurred, as no correction for multiple testing was performed. Based on their and our observations, we suggest that cytokines may lower the pain threshold, but that this effect is likely modulated by one or more other downstream mechanisms. A possible mechanism could be the regulation of pain by glia cells of the CNS [27]. In addition, activation of the vagus nerve by other substances apart from cytokines [13] as well as local production of IL-1 in peripheral tissues and the central nervous system may play an important role.

A limitation to the study is that the group not treated with endotoxin did not receive a venous and arterial catheter, did not receive (pre)hydration solution and did not fast 12 hours before the start of the experiments. Possibly one of these factors resulted in the baseline differences as were found in PPT and EPT. Because paired observations within the same subject were obtained, data were analyzed as change from baseline per subject, and no direct statistical comparisons between the endotoxemia group and non-endotoxemia were made, this should have no influence on the interpretation of the study results. Strong aspects of the present study are the fact that we used a validated, standardized model of systemic inflammation, in which purified endotoxin elicited a pronounced inflammatory response, reflected by high levels of circulating cytokines, even compared with septic patients [8]. Furthermore, time-dependent bias was excluded by the inclusion of a non-endotoxin treated study group. Also, three different domains of QST measurements were used; electrical stimulation, pressure algometry, and cold pressor testing, providing solid evidence that multiple measures of somatic pain perception are altered by systemic inflammation. 

The findings of this and previous studies, indicating a clear relation between inflammation and pain perception, contribute to our understanding of pain in systemically inflamed patients such as those suffering from sepsis. Furthermore this study shows that experimental human endotoxemia offers a valuable model to study inflammation-induced hyperalgesia that may facilitate development of new therapies aimed at modulating inflammation-related pain perception. 

## Conclusions

In conclusion, this study shows that systemic inflammation elicited by the administration of endotoxin to human results in a decreased pressure pain threshold, a decreased electrical pain threshold and decreased tolerance to the cold pressor test. In the absence of a correlation with circulating cytokines, the nature of the link between inflammation and pain sensation needs further study.
